# The Effect of Surface Treatments on the Retentive Strengths of Crowns Fabricated From Polymethylmethacrylate (PMMA) Reinforced With Graphene Nanoparticles After Thermocycling: An In Vitro Study

**DOI:** 10.7759/cureus.64699

**Published:** 2024-07-16

**Authors:** Kyathi K Jathan, Ajay Kumar Gaikwad, Pronob Sanyal

**Affiliations:** 1 Prosthodontics and Crown & Bridge, Krishna Vishwa Vidyapeeth, Malkapur, IND

**Keywords:** thermocycling, nanoparticles, pmma, surface treatment, retentive strength, pull out test, graphene, g-cam

## Abstract

Aim: The study aims to assess the effect of surface treatments by chemical agents on the retentive strengths of crowns fabricated from polymethylmethacrylate (PMMA) reinforced with graphene nanoparticles adhesively bonded to abutments after thermocycling.

Settings and design: In vitro comparative study.

Materials and methods: This study is composed of four groups - one control, one treated with 99% pure etchant acetone solution, one treated with 15 wt% potassium hydrogen fluoride solution, and the last group treated with a combination of both solutions.

Results: The results showed that the mean load in Group A is 228.46±3.16, Group B is 252.57±7.14, Group C is 184.51±6.61, and Group D is 211.03±2.54. The mean score is highest for Group B followed by Group A and Group D, and it is least for Group C. One-way analysis of variance (ANOVA) detected highly significant differences (p<0.01) among the four groups.

Conclusion: It can be concluded that acetone is the best chemical etchant solution for crowns fabricated from G-CAM discs (Graphenano Dental, Graphenano Nanotechnologies, Spain).

## Introduction

Polymethylmethacrylate (PMMA) holds a notable position in dentistry due to its unique characteristics, such as aesthetic appeal, affordability, precise adaptation, lightweight nature, and ease of handling besides its application in denture bases [1]. Despite its advantages, PMMA has some drawbacks such as polymerization shrinkage leading to dimensional changes and inaccuracies in denture bases [2]. Various additives, including glass or carbon fibers, metal fillers, and nanotubes, have been integrated into PMMA to serve as reinforcements [3]. Among these, graphene has attracted considerable research interest in the field of PMMA nanocomposites. Graphene, characterized by a single sheet with one-atom thickness arranged in a honeycomb-like lattice, addresses some of these challenges [4]. Graphene is present in various forms, such as graphene sheets, graphene oxide (GO), and reduced graphene oxide (rGO), which can be functionalized and integrated with polymers to create composites with customized properties. GO in particular exhibits promising properties, including biocompatibility, biodegradability, high strength (Young’s modulus of Y~1.0 TPa), antimicrobial-adhesion characteristics, flexibility, and transparency [5].

A new computer-aided design/computer-aided manufacturing (CAD/CAM) material, namely G-CAM, was recently introduced by Graphenano Dental by Graphenano Nanotechnologies, Spain. It consists of CAD/CAM discs containing PMMA reinforced with GO nanoparticles [5]. The graphene nano-reinforced biopolymer G-CAM disc, specially designed for permanent dental structures, is available in different shades that have an extremely natural aesthetic appearance, resolving all the mechanical, physicochemical, and biological failures of the rest of the materials currently used in the sector. However, with the use of these crowns clinically, debonding in less than nine months of use was noted intraorally [6].

What was lacking was a chemical for surface treating the intaglio surface of the crowns to increase the retentive strengths of these crowns [6]. Literature lacks the studies where the surface treatments on graphene crowns have been conducted with the objective of increasing surface retention. Simulation of the oral environment can help determine the longevity and efficiency of the performance of these crowns under the influence of the oral environment, which is lacking in the literature. With the above accordance, this study is directed towards analyzing the improvement of the retentive strengths of the GO reinforced crowns by surface treating the intaglio surface of these crowns with chemical reagents in an attempt to prevent debonding during use and hence improve the overall clinical performance of these crowns.

## Materials and methods

**Figure 1:**
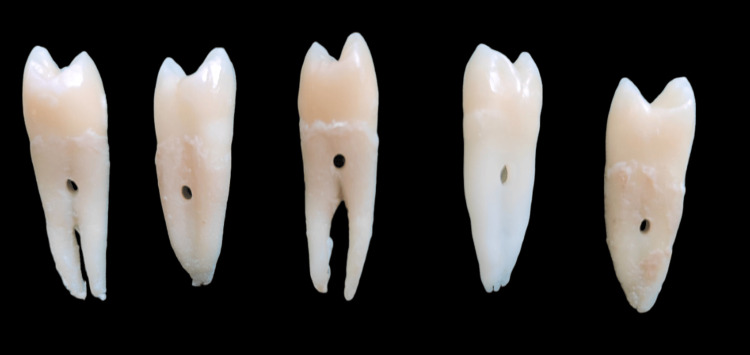
Retentive elements drilled into the roots of the premolar teeth

A sample size of 40 dental crowns was determined by the software G*Power 3.1.9.2 (Heinrich Heine University Düsseldorf, Düsseldorf, Germany) and divided into four subgroups of 10 samples each. The 40 non-carious maxillary premolars that were extracted for periodontal or orthodontic purposes were collected and cleaned of gross debris in sodium hypochlorite solution (1:10) and kept in distilled water (Vitzee Chemicals, India) to maintain hydration. Retentive elements were drilled into the roots of the premolar tooth to ensure proper retention and to avoid dislodgement during testing (Figure 1).

**Figure 2:**
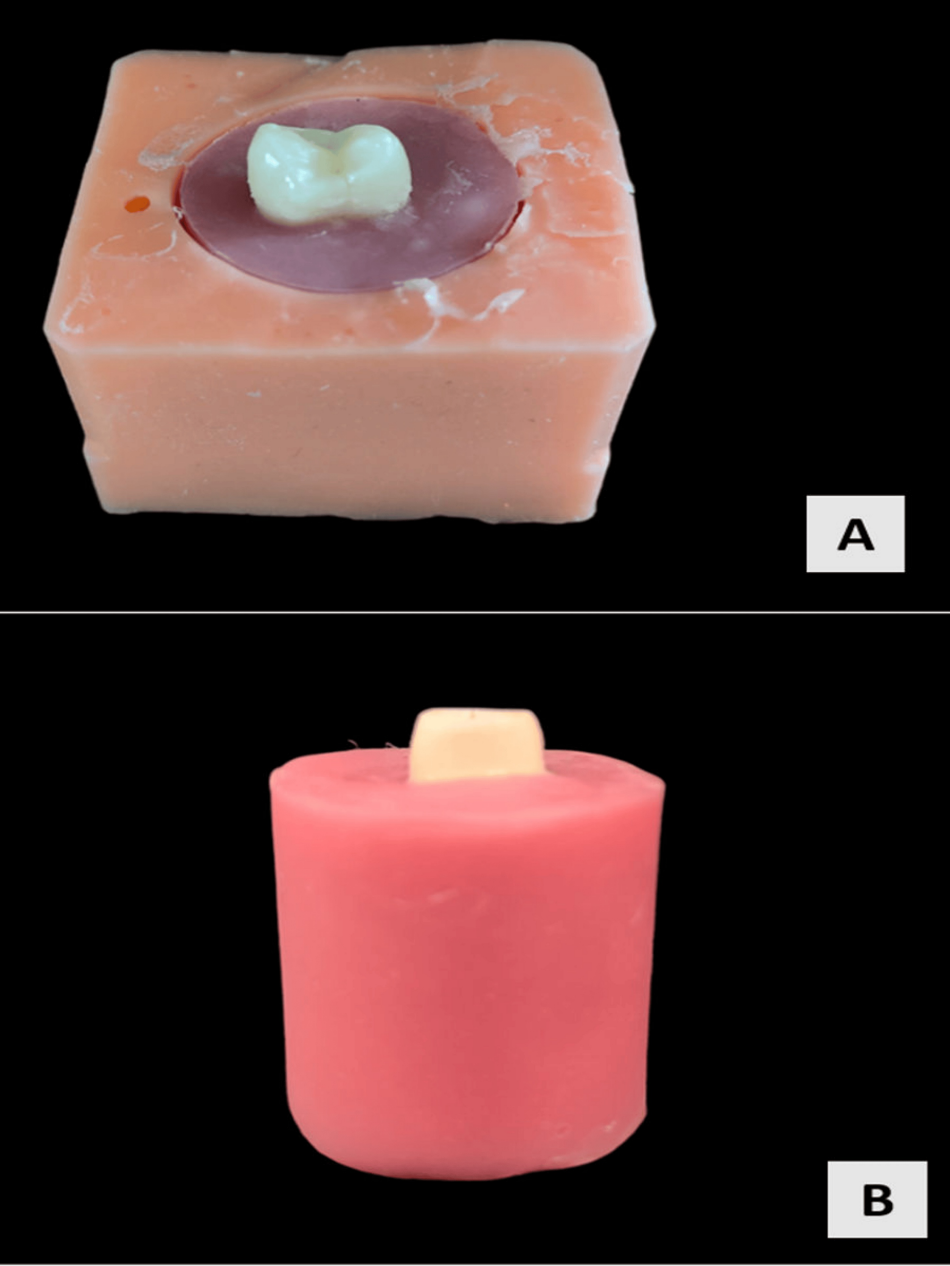
(A) Extracted premolars positioned in the acrylic block in the mold; (B) Tooth preparation done on the mounted samples

Acrylic resin blocks were created using a silicon mold that was 20 mm long, 20 mm wide, and 26 mm thick prepared according to the International Organization for Standardization (ISO) standardization (ISO1567:1999). The mold was filled with autopolymerizing acrylic resin after being mixed according to the manufacturer's guidelines. The extracted maxillary premolars were then positioned in the center of the material so that only the crown portion of the tooth was visible above the surface (Figure 2A). Tooth preparation of the crowns of the abutment teeth samples was done by one operator to maintain uniformity of the preparation. The occlusal surface was prepared flat and the height of preparation from the cementoenamel junction (CEJ) was 4 mm with a uniformly maintained taper of 6° (Figure 2B).

**Figure 3:**
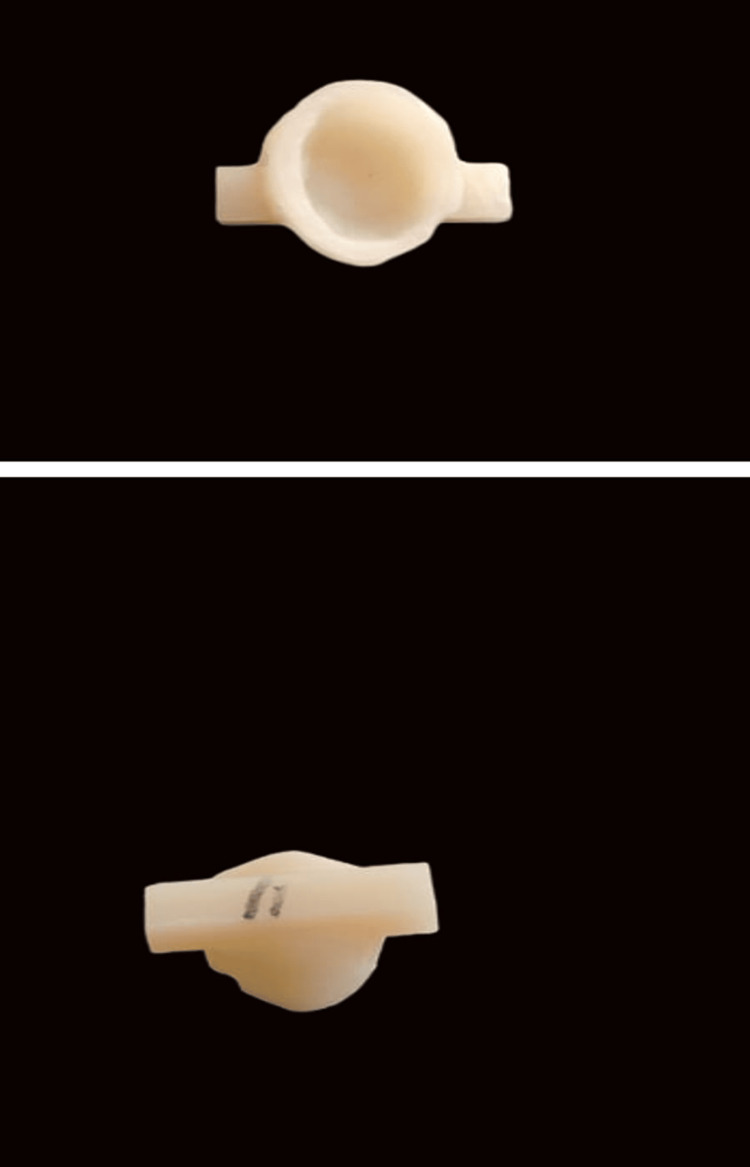
Milled G-CAM premolar crown with retentive bar

The prepared tooth samples were scanned and computer-aided designing was carried out in the Exocad software (Exocad GmbH, Darmstadt, Germany) to determine the design of the crown with retentive features. This design then went ahead with subtractive milling of the crowns from the G-CAM discs (Graphenano Dental, Graphenano Nanotechnologies, Spain). To aid in the retentive strength testing in a universal testing machine, an occlusal bar was incorporated in the computer-aided design of the crowns to allow pull-out testing of these crowns (Figure 3).

Following this, the two etchant solutions that were to be used for surface treating the intaglio surface of the crowns were prepared. A 99% pure etchant acetone solution (Loba Chemie Pvt. Ltd., Mumbai, India) was readily available and was used in the same concentration without any manipulation. A 15 wt% potassium hydrogen fluoride powder was laboratory prepared by mixing it with carboxymethylcellulose powder (Loba Chemie Pvt. Ltd., Mumbai, India) in medium viscosity in distilled water to make its consistency appropriate to be used for surface treatment of the crowns. All intaglio surfaces of the 40 crowns were subjected to sandblasting with 50 µm aluminum oxide particles for 45 seconds at 0.2 MPa at 45° angle from a distance of 10 mm and subsequently rinsed off with ethyl alcohol to get rid of the residues. This protocol was provided by the manufacturer, and therefore, all crowns underwent the same procedure before any other surface treatment was applied.

The specimens in Group A (Blue) were treated as control and no surface treatments were administered besides sandblasting with 50 µm aluminum oxide particles for 45 seconds.

The intaglio portion of the crowns of specimens in Group B (Green) was treated with 99% pure etchant acetone solution. The solution was applied in one direction forming a thin layer with the help of an applicator tip and then dried for 30 seconds with an air spray.

The intaglio surface of the crowns of specimens in Group C (Purple) was treated with the 15 wt% solution of potassium hydrogen fluoride. The solution was applied with an applicator tip, kept for 180 seconds, and then rinsed off and dried with air spray.

The intaglio surface of the crowns of specimens in Group D (Red) was first treated with 99% pure etchant acetone solution and then dried after 30 seconds, followed by 15 wt% solution of potassium hydrogen fluoride that was applied with an applicator tip, kept for 180 seconds, and then rinsed off and dried with air spray.

**Figure 4:**
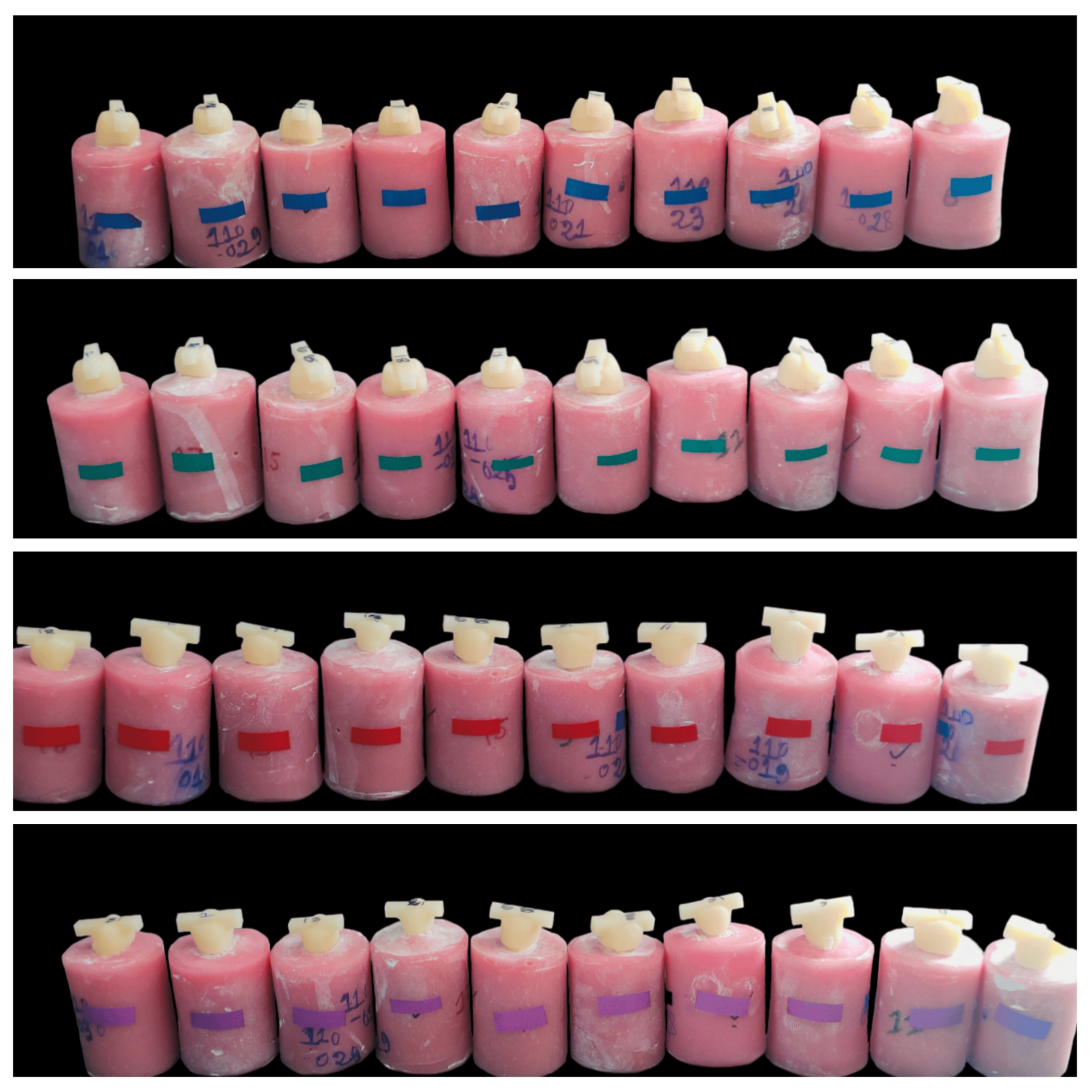
Group A (Blue); Group B (Green); Group C (Red); Group D (Purple)

The crowns were then luted on the prepared abutment teeth specimens with self-adhesive resin cement (PANAVIA SA Cement Universal, Kuraray Noritake Dental, Japan) by correctly adhering to all the cementation protocols and then distributed into groups (Figure 4).

**Figure 5:**
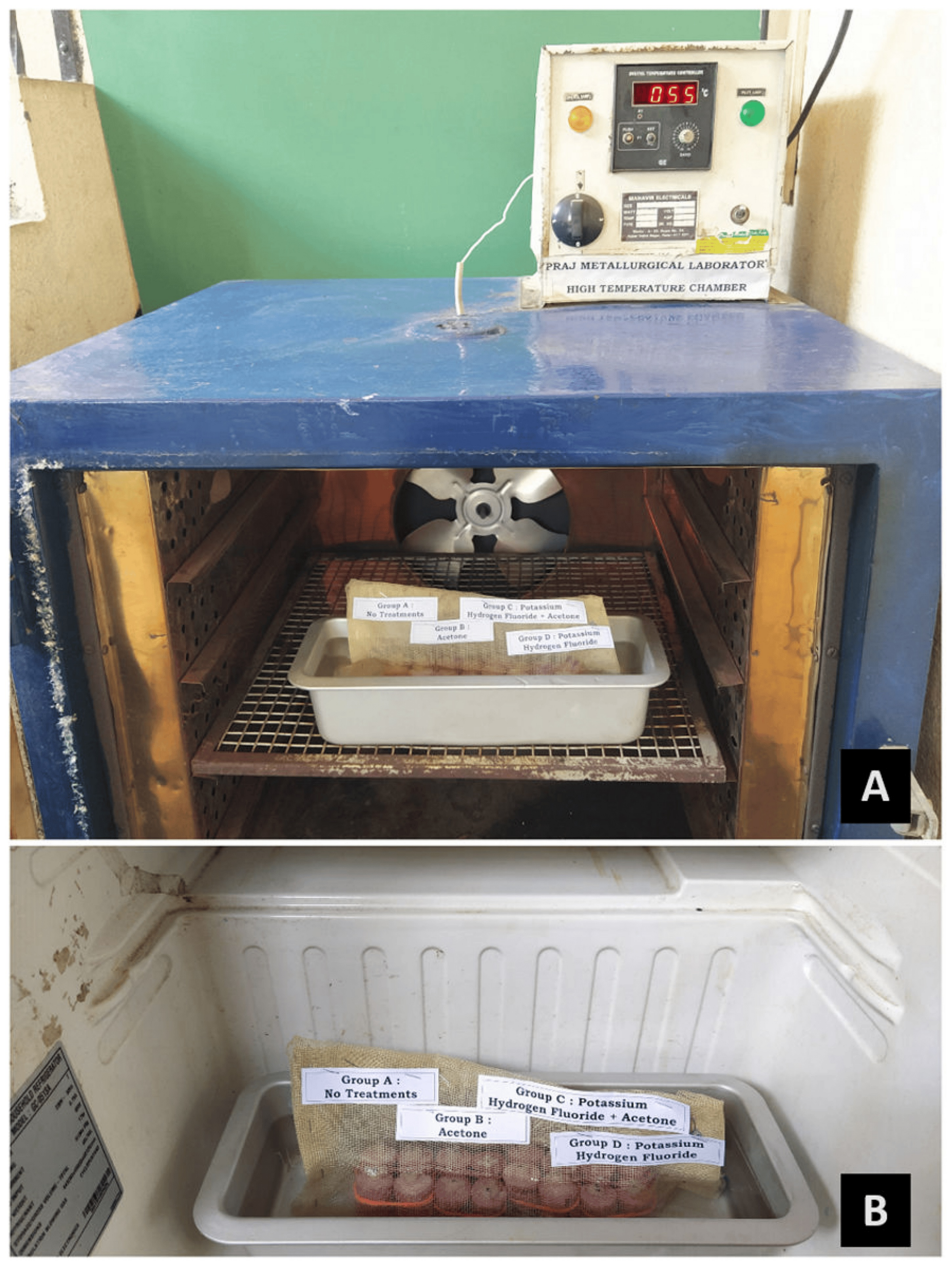
(A) Storage in the water bath; (B) Thermocycling at 5℃ and 55℃

The specimens, according to the group they belong to, were stored in a water bath at 37°C for one week (Figure 5A), following which they were subjected to thermocycling for 10,000 cycles between 5℃ and 55℃ with a dwell time of 30 seconds (Figure 5B). This simulated the intra-oral performance of the crowns for one year.

**Figure 6:**
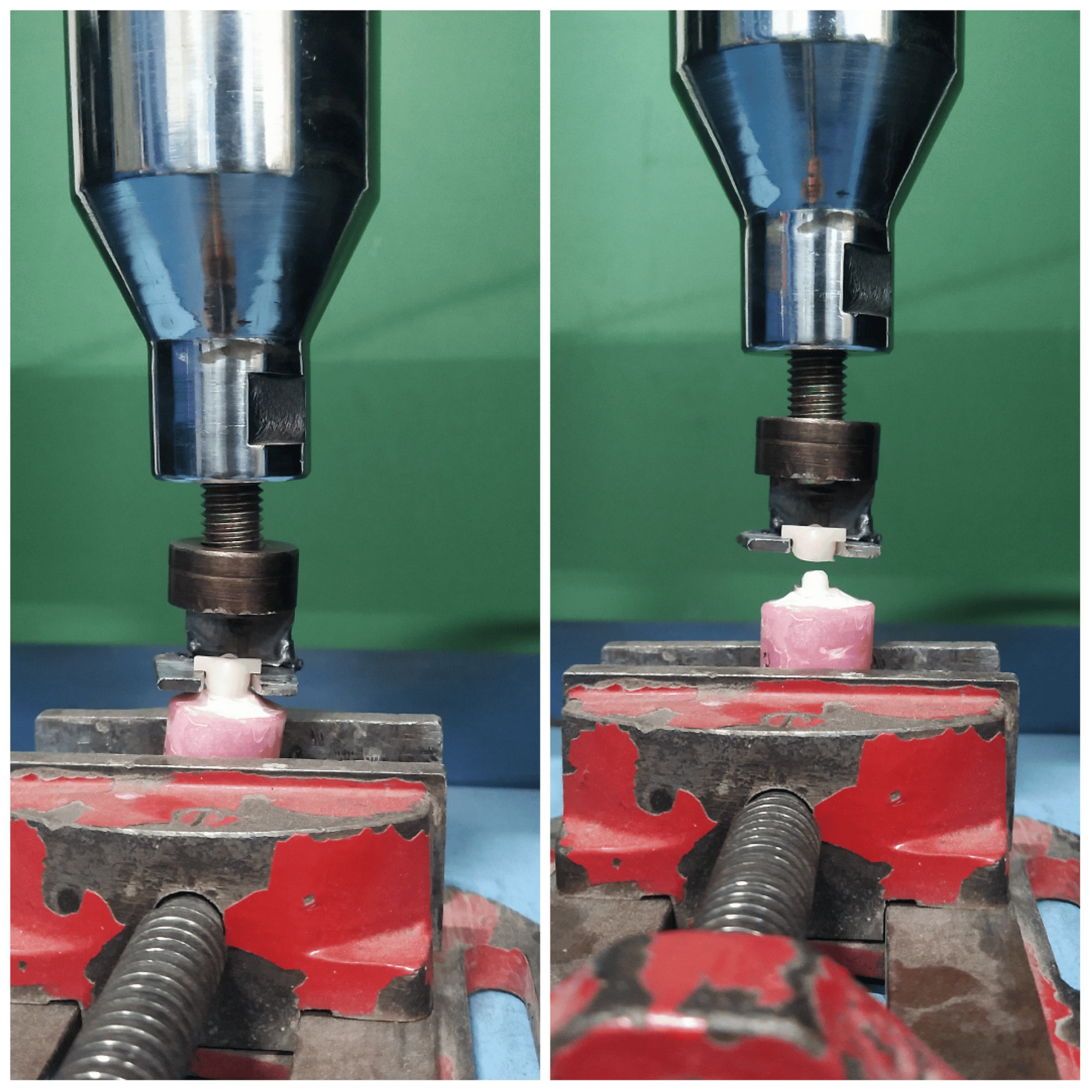
Samples assembled for pull-out testing in the universal testing machine

The thermocycled specimens were then placed on a universal testing machine (Model: UNITEST-10; ACME Engineers, Pune, India), and they underwent pull-out testing at a crosshead speed of 5 mm/min until the crown was debonded from the tooth-mounted in acrylic resin (Figure 6).

This process was carried out for all 40 specimens, with the attachment secured to the occlusal bar designed on the crowns. The maximum load at which the crown separated from the prepared tooth was recorded and retentive strength was calculated with the following formula:

Retention strength (N) = Fracture load (N)/Bond area (surface area measured) (mm^2^)

**Figure 7:**
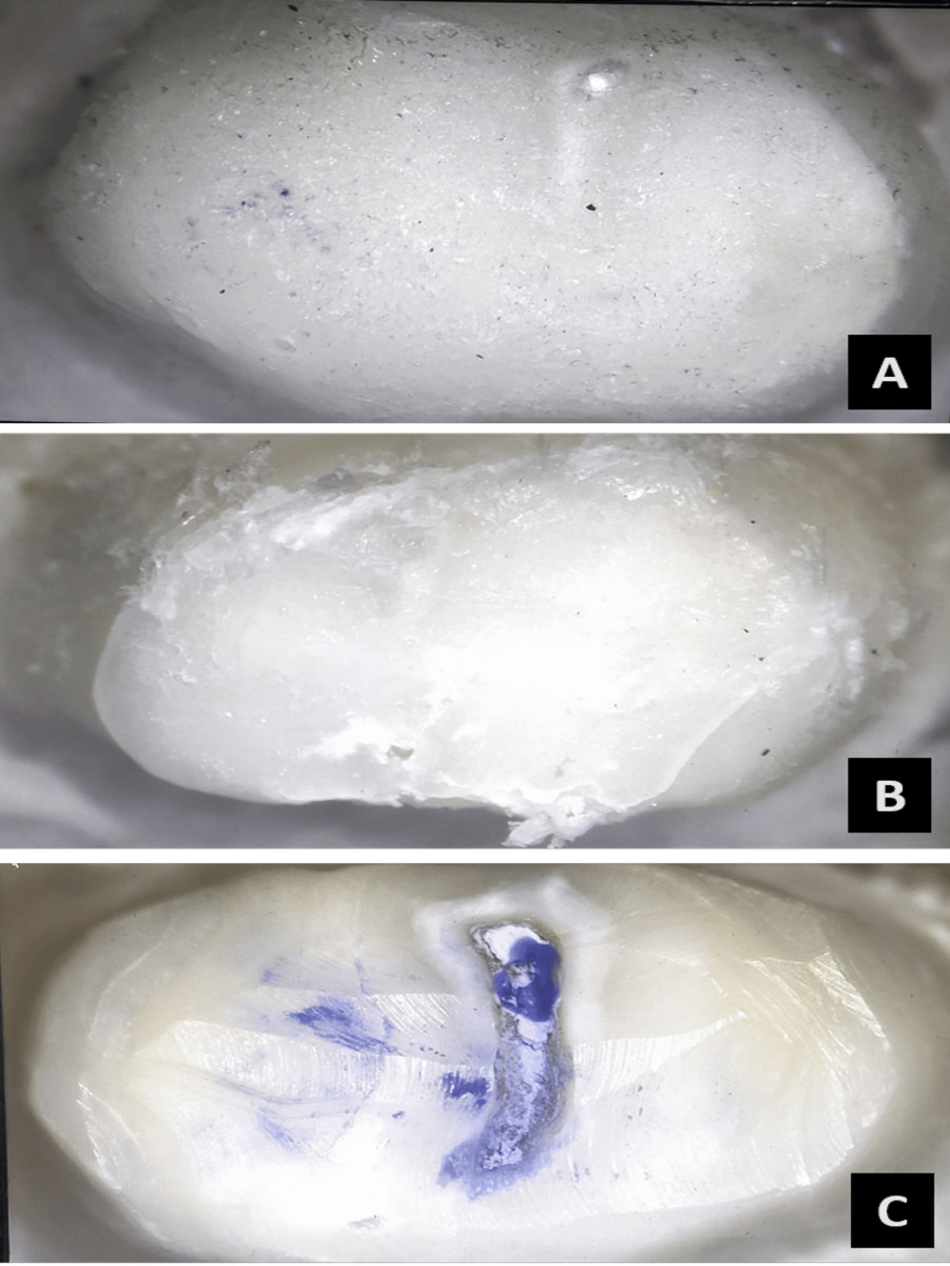
(A) Adhesive failure; (B) Cohesive failure; (C) Mixed failure

The values were noted and the intaglio surface of the debonded crowns and the surface of the abutment teeth were studied under a stereomicroscope (Model: XTL 3400E; Wuzhou New Found Instrument Co. Ltd., China) at 40X magnification to identify the type of failures that were classified under adhesive, cohesive and mixed type of failures (Figure 7).

After determining the type of failure, the intaglio surface of crowns from all four groups was scanned under the scanning electron microscope (FEI Quanta 200, The Netherlands) to assess the structural surface changes that occurred after treatment with the chemical reagents. The surface morphology was studied to look for micro-irregularities caused by the etching of the crowns under 100X, 500X, and 1000X magnification. 

## Results

Descriptive statistics for retentive strengths were presented as mean ± standard deviation (SD). An inter-group comparison was conducted to compare fracture resistance among the four groups using one-way analysis of variance (ANOVA), followed by the post hoc Tukey test for pairwise comparisons. The mode of failure among the four groups was assessed using the Chi-square test. Statistical analysis was performed at a 95% confidence level. In the above tests, p-value ≤0.05 was taken to be statistically significant. All analysis was performed using IBM SPSS Statistics for Windows, version 26.0 (IBM Corp., Armonk, USA).

Table 1 shows the comparison of fracture resistance among four groups. The mean load in Group A was 228.46±3.16, Group B was 252.57±7.14, Group C was 184.51±6.61, and Group D was 211.03±2.54. The mean score was highest for Group B, followed by Group A and Group D, and least for Group C. One-way ANOVA detected a highly significant difference (p<0.01) among the four groups.

**Table 1:** Comparison of fracture resistance among all the four groups

	N	Mean	Std. Deviation	Std. Error	95% Confidence Interval for Mean		Minimum	Maximum	F	p-value
					Lower Bound	Upper Bound				
Group A	10	228.4600	3.16340	1.00036	226.1970	230.7230	223.70	232.50	295.67	0.000 HS
Group B	10	252.5700	7.14890	2.26068	247.4560	257.6840	245.50	264.20		
Group C	10	184.5100	6.61655	2.09234	179.7768	189.2432	175.40	190.30		
Group D	10	211.0300	2.54299	.80416	209.2109	212.8491	207.60	214.10		

A highly significant difference was observed between all pairs with a p-value less than 0.01. (p<0.01) and SD of 2.3 according to the post hoc Tukey test, along with the distribution of mode of failures among four groups as shown in Table 2.

**Table 2:** Determination of the type of failure after retentive strength testing

			Mode of Failure			Chi-square	p-value
			Adhesive	Cohesive	Mixed		
Group	Group A	Count	10	0	0	13.6	0.03 S
		% within Group	100.0%	0.0%	0.0%		
	Group B	Count	2	5	3		
		% within Group	20.0%	50.0%	30.0%		
	Group C	Count	5	5	0		
		% within Group	50.0%	50.0%	0.0%		
	Group D	Count	5	3	2		
		% within Group	50.0%	30.0%	20.0%		

In Group A, the mode of failure in all samples was adhesive (100%). In Group B, 20% of failures were adhesive, 50% of samples underwent cohesive failure, and 30% were mixed failures. In Group C, 50% of failures were of the adhesive type and 50% were cohesive. In Group D, 50% of failures were of the adhesive type, 30% were of a cohesive nature, and 20% were mixed. A significant difference was observed in the mode of failures among the four groups when the Chi-square test was applied (p=0.03).

**Figure 8:**
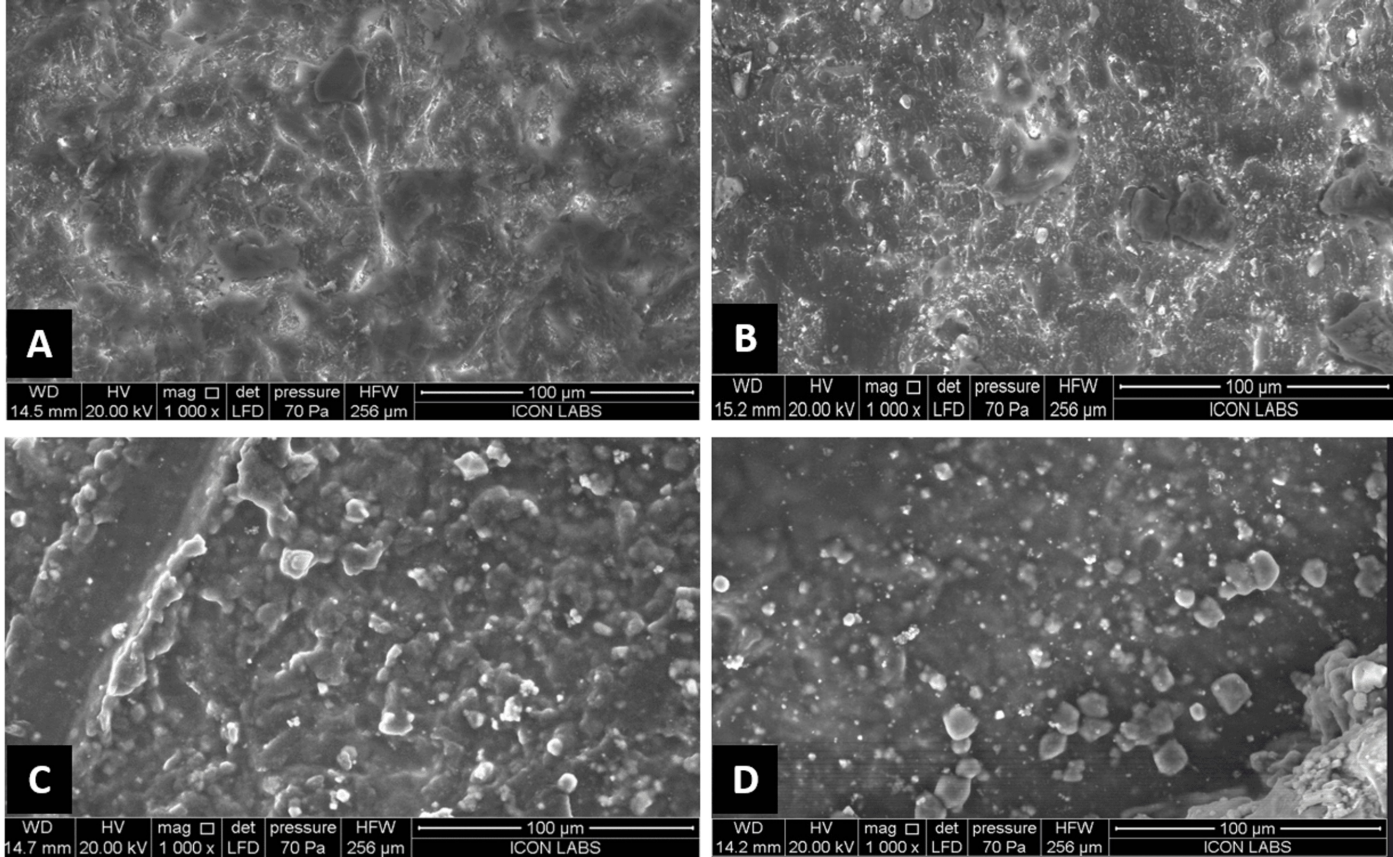
(A-D) Scanning electron microscopy analysis of Groups A, B, C, and D under 1000X magnification

The scanning electron microscopy (SEM) analysis of the samples in Group A, which is the control group, showed a surface abraded by 50 microns of alumina particles depicting natural undercuts and uniform irregularities (Figure 8A). The images of Group B, which is the 99% pure etchant acetone group, showed an altered surface with significant grooving and sharp edges along with numerous pits at various locations on the surface (Figure 8B). Groups C and D showed clusters of sintered particles unaffected by the abrasive. Smoothening out of the irregular pits and craters as compared to the sandblasted surface in the control group was seen with irregularities blocked out, resulting in an even surface with few pits and clusters (Figures 8C-8D). 

## Discussion

PMMA is the preferred material for the fabrication of complete dentures; however, it falls short of being an ideal material for meeting the mechanical requirements of a fixed definitive prosthesis [6]. The data indicates that incorporating high-elastic modulus graphene into a low modulus polymeric matrix can result in significant reinforcement. Lee et al. and Agarwalla et al. examined the mechanical property in question and observed that the inclusion of graphene led to an increase in the hardness of the resin [7]. This suggests that adding graphene can effectively strengthen the overall mechanical properties and performance of polymeric materials, making them suitable for various applications where enhanced mechanical characteristics are beneficial [8,9]. The inclusion of GO can notably enhance the tribological performance of PMMA composites, primarily due to its favorable compatibility, dispersibility, and reinforced interface with the polymer matrix [10,11]. Incorporating graphene structures leads to strong chemical interactions within the matrices. Achieving a uniform dispersion of graphene structures ensures an even distribution of stress, significantly boosting the mechanical strength of the nanomaterial. Ionescu et al. mentioned that the presence of active functional groups on the surface of GO promotes favorable compatibility with the PMMA matrix [12].

In this study, a newly introduced material for definitive prostheses was examined by testing the effect of surface treatments on bond strength through pull-out testing. The chemical inertness, insolubility, and resistance to water and saliva of the G-CAM discs make them well-suited for dental applications [13]. This study focused on the use of this versatile material in crown fabrication as it is newly introduced and gaining popularity among dental practitioners. Upon further clinical-based examinations and clinician experiences, it was found that clinically these crowns experienced debonding in less than nine months of use. This highlights the importance of assessing the material's retentive strength.

Another study by Palacios et al. examined resin cement and assessed the removal strength of custom-made zirconium oxide ceramic copings fabricated using CAD/CAM technology for permanent teeth [14]. The retentive strengths recorded were 647.78 N for RelyX Unicem, 652.68 N for Panavia, and 782.04 N for RelyX Luting. However, the values reported in that study were higher than those observed in the present study, wherein the mean retentive strength is 252.56 N. No data was available regarding the retentive strength measurement in G-CAM crowns, that is crowns fabricated from PMMA reinforced with graphene. Hence, data from similar studies with different materials were taken as a reference for retentive strength values for this study, wherein the highest mean is 252.56 N [15]. The chemicals were chosen in accordance with the literature that supports the action of these chemicals on the principal contents of the G-CAM disc, mainly PMMA and graphene nanoparticles.

To efficiently perform pull-out tests and measure the retentive strengths of the samples after surface treatments with the specified reagents, an occlusal bar was incorporated into the crown design using CAD software before milling the crowns [16]. A detailed analysis of the data revealed that the highest retentive strength was recorded in samples from Group B, which were treated with 99% pure acetone. Samples from Group D, which were treated with both acetone and potassium hydrogen fluoride, showed retentive strength values between those of Group B and Group C, indicating fairly acceptable results. These findings suggest that the combination of acetone and sandblasting can induce surface alterations on the intaglio surface of G-CAM crowns, enhancing retentive strengths even after thermocycling, which simulates oral conditions. This is supported by the SEM image, which shows significant pits and micro-irregularities in the crown's normal structure in the control group. This aligns with the mechanism of superficial crack propagation and the formation of numerous pits, approximately 2 µm in diameter, which allow the luting agent to infiltrate these micro-irregularities adequately, thereby successfully increasing the crowns' retentive strength.

When considering the use of potassium hydrogen fluoride, it was found that this reagent tended to block the micromechanical irregularities created on the intaglio surface of the crowns. As a result, it could not withstand the forces of withdrawal during the pull-out testing after thermocycling. SEM images revealed that the irregularities were reduced compared to both the control group and the acetone group, suggesting that the molecules in the 15 wt% potassium hydrogen fluoride solution may have filled in these pits, resulting in a smoother surface.

The current laboratory study was conducted without the presence of oral conditions such as gum bleeding or saliva contamination during the cementation process. These factors could influence the retention strength of graphene-reinforced crowns in clinical settings. Future investigations could explore these factors by introducing known contaminants during testing. Additionally, longer testing periods could be considered to assess the impact of occlusal forces, parafunctional habits, toothbrushing, and chewing on the crowns over time. As these factors can vary among individuals, in vivo experiments are recommended to validate the findings of this in vitro study.

## Conclusions

Within the limitations of this study, the following conclusions can be drawn. The highest retentive strength values were observed in samples treated with 99% pure acetone solution. Crowns treated with this etchant showed superior bonding, evidenced by maximum cohesive failures and a few mixed and adhesive failures, indicating a strong bond between the adhesive and the substrate. In contrast, using 15 wt% potassium hydrogen fluoride for surface treatment of G-CAM crowns is not recommended due to poor retentive strength caused by the blocking of micro-irregularities and surface pits created by sandblasting. Crowns treated with both 99% pure acetone and 15 wt% potassium hydrogen fluoride exhibited moderately satisfactory results, but this dual reagent method is not recommended due to its complexity and inconvenience. Overall, applying the 99% acetone solution is highly recommended for improving the clinical performance of crowns made from PMMA reinforced with graphene nanoparticles and luted with resin cement.
